# Preparation of Small-Particle and High-Density Cobalt Carbonate Using a Continuous Carbonate Precipitation Method and Evaluation of Its Growth Mechanism

**DOI:** 10.3390/ma12203394

**Published:** 2019-10-17

**Authors:** Miaomiao Guo, Xiaoli Xi, Sen Li, Chenwei Li, Zuoren Nie, Kaihua Xu

**Affiliations:** 1 National Engineering Laboratory for Industrial Big-data Application Technology, Beijing University of Technology, Beijing 100124, China; guo6miao2@emails.bjut.edu.cn (M.G.); xixiaoli@bjut.edu.cn (X.X.); 2 Chinese National WEEE Recycling Engineering Research Center, Jingmen GEM New Material Co., Ltd., Jingmen 448124, China; listen178@126.com (S.L.); lichenwei@gem.com.cn (C.L.); xukaihua@gem.com.cn (K.X.)

**Keywords:** cobalt carbonate, small particle, high density, growth mechanism

## Abstract

Spherical CoCO_3_ powder with a small particle size and high density was successfully prepared using a continuous carbonate liquid precipitation method with a raw material of cobalt chloride solution, a precipitant of NH_4_HCO_3_, and without a template. The effects of the concentration of ammonium carbonate, process pH, and feeding rate on the tap density and apparent density of cobalt carbonate were investigated. It was found that the apparent and tap density values of 4.4 µm of cobalt carbonate were 1.27 g/cm^3^ and 1.86 g/cm^3^, respectively, when the initial concentration of NH_4_HCO_3_ solution was 60 g/L, the pH was 7.15–7.20, and the feeding rate of cobalt chloride was 2 L/h. The anisotropic growth process of the crystal lattice plane of CoCO_3_ under the aforementioned optimal conditions were studied. The results demonstrated that the crystal grew fastest along the (110) facet orientation, which was the dominant growth surface, determining the final morphology of the primary particles. The scanning electron microscopy (SEM) and high-resolution transmission electron microscopy (HR-TEM) results demonstrated that the primary particle morphology of the cobalt carbonate was a nanosheet. The unit cell of cobalt carbonate, of a hexagonal structure in the horizontal direction, grew horizontally along the (110) facet orientation, while 20–35 unit cells of the carbon carbonate were stacked along the c-axis in the thickness direction. Finally, the sheet-shaped particles were agglomerated into dense spherical secondary particles, as presented through the crystal re-crystallization model.

## 1. Introduction

As an important chemical material, cobalt carbonate belong to the hexagonal system and $D_{3d}^{6}$ space group ($R\bar{3}c$) with lattice parameters of a = 4.633 Å, b = 4.633 Å, c = 14.998 Å, α = 90°, β = 90°, and γ = 120° (JCPDS No. 78-0209) [1]. It is widely utilized in ceramic industry colorants [2], organic industrial catalysts [3], lithium ion battery cathode materials [4,5,6,7,8], and precursors [9,10,11,12], including those precursors for the cemented carbides industry [13,14,15]. The synthesis method of cobalt carbonate include the hydrothermal process [13], solvothermal process [16], as well as batch or continuous methods of the carbonate or bicarbonate precipitation process [17,18,19]. Compared to the hydrothermal process and solvothermal process, the solubility product, namely *K_sp_*, of carbonate or bicarbonate precipitation processes is 1.4 × 10^−13^, presenting a relatively high precipitation efficiency. Furthermore, the precipitation process is widely industrialized in the precursor field for manufacturing the used 3C lithium ion battery to prepare the cobalt oxide, aluminum-doped cobalt oxide, and lithium cobalt oxide due to the fact that the raw materials are cheap and easy to obtain, while the preparation technology is simple and the production efficiency is high [20,21,22,23].

The theoretical density of cobalt carbonate is 4.13 g/cm^3^ [8], while the tap density of the powder is much lower than the theoretical value due to the influence of pores and impurities in the middle of the powder. However, most of the commercial cobalt carbonate product possesses a tap density below 1.0 g/cm^3^ [24]. Tap density (TD), presenting the density when cobalt carbonate powder is closely stacked, is a significant parameter for the lithium ion battery material. The TD of cobalt carbonate will directly affect the TD of lithium cobalt oxide and cobalt oxide in the lithium ion battery and the production efficiency, meanwhile, it will indirectly impact the capacity density of the lithium ion battery. Therefore, the surface loose cobalt carbonate with a low tap density could not meet the requirement of the high-performance lithium ion battery precursor. 

Although this material has wide industrial applications in the lithium ion battery field, however, only a few investigations focused on the influence of the batch process on the performance of specific physicochemical parameters, such as particle size, morphology, impurities, and so on [16]. Meanwhile, classic crystallography only considers the nucleation process, growth process, and the supersaturation driving force through crystallization thermodynamics [25,26]. However, there are quite a few literature reports about how the continuous precipitation technology affects the density of cobalt carbonate [4]. Even so, none of research reported on the agglomeration of continuously synthesized small-particle and high-density cobalt carbonate and the correlative mechanisms. 

In this paper, we present a study on the continuous liquid phase precipitation process for the preparation of cobalt carbonate with a small particle size and high tap density. According to the X-ray diffraction (XRD) data processing using the Scherrer equation, the average crystal size variation in different facet orientations and the predominant growing facets during the entire crystallization were investigated. Moreover, through scanning electron microscopy (SEM), high-resolution transmission electron microscopy (HR-TEM), and a detailed analysis of the unit cell, the growing process of primary particles and the agglomeration mechanism of secondary particles were studied. The results could increase the preparation comprehension of cobalt carbonate through continuous precipitation, as well as the preparation of lithium cobalt oxides with high energy capacity. 

## 2. Experimental

### 2.1. Material Preparation

The CoCl_2_ solution was obtained from Jingmen GEM New Material Co., Ltd. (Jingmen, China). Ammonium bicarbonate solution was mixed with industrial ammonium bicarbonate, which was purchased from Hubei Sanning Chemical Co., Ltd. (Zhijiang, China).

CoCO_3_ powders were prepared through a continuous overflow carbonate liquid precipitation method. Concentrations of 200 g/L of NH_4_HCO_3_ solution and 130 g/L of cobalt chloride solution were pumped into a reactor kettle under vigorous stirring. At the base concentration of NH_4_HCO_3_ of 60 g/L (except the base solution concentration experiment) and the initial flow of CoCl_2_ of 2 L/h (except the cobalt flow experiment), the pH value was controlled within a small region through the flow change of NH_4_HCO_3_. When the particles’ size reached the target value of 4.4 µm, the slurry solution within the reactor kettle was washed several times in the centrifuge and dried at 105 °C for 10 h to produce the cobalt carbonate samples. During the mechanism investigation experiments, the slurry solution within the reactor kettle under different crystallization times was washed several times and dried at 105 °C for 10 h to produce the cobalt carbonate samples.

### 2.2. Characterization of CoCO_3_

Scanning electron microscopy (SEM) was done using a Quanta FEG 250 (Thermo Fisher Scientific, Waltham, MA, USA), high-resolution transmission electron microscopy (HR-TEM) was performed with a JEM-2100 instrument (JEOL Ltd., Tokyo, Japan) operated at 200 kV. The samples were ultrasonically suspended in the ethanol solvent, and one or two droplets of this slurry were deposited on a copper grid. 

Structure information of the material were obtained through X-ray diffraction experiments, which were conducted using a Lab 6100 X-Ray Diffractometer (Shimadzu Corporation, Tokyo, Japan) with a Cu Kα source (*λ* = 1.5418 Å, a range of 15°–75°, 4°/min). The crystallite size was calculated using the Scherrer equation (Equation (1)):

(1) $$K_{hkl}=k\lambda /B\cdot cos\theta$$

where, *K_hkl_* is the grain size (nm) along the [hkl] direction, *λ* is the wavelength of the radiation (*λ* = 1.54056 Å), *k* is the Scherrer constant taken to be 0.89, *B* is the full width at half maximum (FWHM), and *θ* is the diffraction angle.

Tap density (TD) and apparent density (AD) were obtained using FZS4-4B tap density (Central Iron & Steel Research Institute, Beijing, China) and FL4-1 apparent density detectors (Central Iron & Steel Research Institute, Beijing, China). The AD or random loose packing of the powders was determined using the standard [27] cup and funnel method. The size distribution was found using a Mastersizer 2000E Laser particle analyzer (Malvern Panalytical, Worcestershire, UK).

## 3. Results and Discussion

### 3.1. Density of CoCO_3_

#### 3.1.1. Effect of Concentration of NH_4_HCO_3_ on the Density of CoCO_3_

The principle behind preparing CoCO_3_ through a liquid precipitation method was to achieve a concentration of Co^2+^ and CO_3_^2−^ exceeding the substance’s precipitate solubility product under certain condition ([Co^2+^][CO_3_^2−^] > *K_SP_*(CoCO_3_)). Classic crystallography divided the crystallization into two stages, namely the nucleation and growth. The nucleation stage, which determined the initial particle size of the crystal, and hence influence the density of the crystal, as well as other physicochemical parameters, was the fundamental step for the following growth. Furthermore, the base concentration of NH_4_HCO_3_ was the key factor for nucleation.

**Figure 1:**
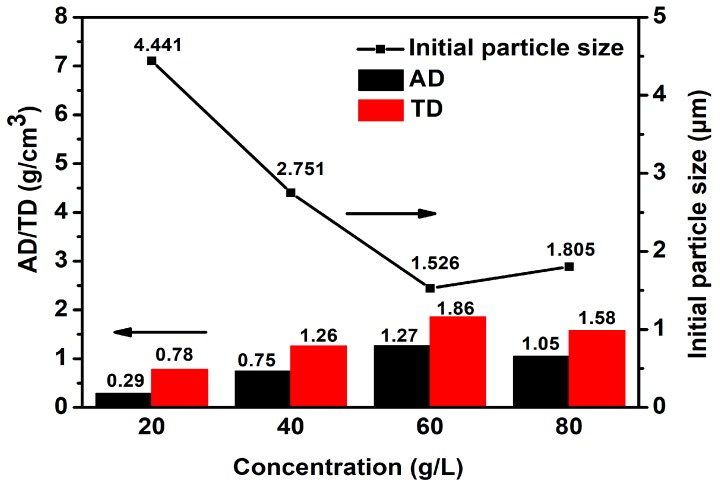
Variation of initial particle size, and the apparent density (AD) and tap density (TD) with concentration changes of the base solution.

The effects of the base concentration on the initial particle size, apparent density (AD), and tap density (TD) of the CoCO_3_ were investigated. The result, as presented in Figure 1, shows that the initial particle size decreased in the nucleation stage from 4.441 µm to 1.526 µm when the base concentration increased from 20 g/L to 60 g/L. Meanwhile, both the AD and TD increased to the maximum values of 1.27 g/cm^3^ and 1.86 g/cm^3^, respectively. Subsequently, the initial particle size increased, while both AD and TD decreased along with the base concentration increase. 

Since the cobalt flow was set at a certain value, the higher the CO_3_^2−^ ion concentration was, the higher the value of [Co^2+^][CO_3_^2−^] was in the reaction system. This led to higher supersaturation (Equation (2)). Furthermore, according to the Gibbs–Thompson equation (Equation (3)), the increased supersaturation would lead to a lower radius of critical nucleation, leading the nucleation to become significantly easier, consequently leading to additional crystal seeds of lower crystal size under the same metal content condition. However, in the 60 g/L to 80 g/L range, the particle size increased along with the base concentration increase of NH_4_HCO_3_. The main reason for the increased particle size was that the extremely high pH, caused by the NH_4_HCO_3_ concentration, increased the amount of free ammonia through decomposition, which resulted in the complex cobalt ion increase within the solution. Due to the complex cobalt ion increase, the [Co^2+^], directly forming precipitates with carbonates, significantly decreased in amount, which led to the value reduction of [Co^2+^][CO_3_^2−^] in the reaction system. Consequently, the supersaturation, the nucleation speed, and the amount of crystal seeds decreased, which drove the particle size of nucleation to become much higher under the same metal content condition. The lower the particle size of nucleation was, the higher the space for crystal arrangement and growth was. This proved favorable for the cobalt carbonate crystal accumulation. Subsequently, the AD and TD would increase through the particle size decrease of nucleation.

(2) $$S=\sqrt{Cm*Cs/Ksp\left(ms\right)}$$

where, *S* is supersaturation; *C_m_* is the concentration of metal ions; *C_s_* is the precipitant ion concentration; *K_sp_* (ms) is the precipitation solubility product constant.

(3) $$ln\frac{C}{C_{s}}=\frac{2\sigma \cdot M}{RT\rho \cdot r_{crit}}$$

where *C* is the concentration of the solution, *C_s_* is the supersaturation, *σ* is the surface tension, *M* is the molar mass, *R* is the thermodynamic constant, *T* is the thermodynamic temperature, *ρ* is the density, and *r_crit_* is the critical radius.

#### 3.1.2. Effect of pH on the Density of CoCO_3_

In the previous section, it was demonstrated that the pH affected the free ammonia, which was decomposed by NH_4_HCO_3_, directly affecting the concentration of [Co^2+^]. In addition, the pH also affected the concentration of [CO_3_^2−^] combined with [HCO_3_^2−^], indirectly influencing the supersaturation in the reaction system. Furthermore, it also affected the crystal plane growth orientation, the initial particle size, and the secondary accumulation model [26]. Therefore, the AD and TD of the CoCO_3_ particle was indirectly affected finally.

**Figure 2:**
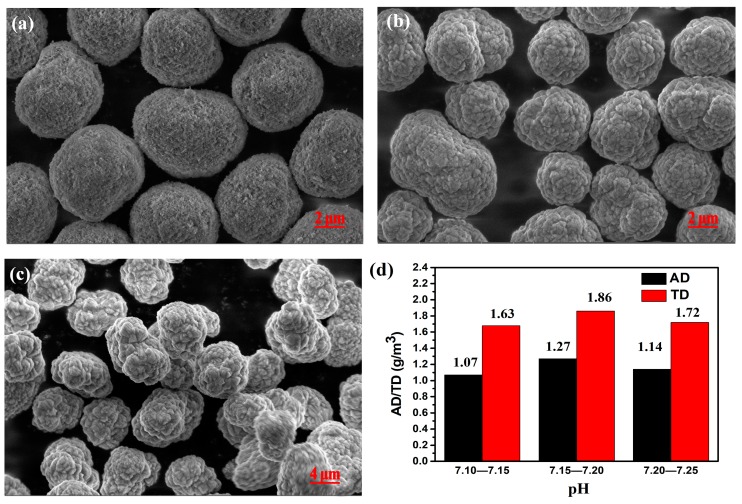
SEM images of CoCO_3_ prepared under all pH ranges: (**a**) 7.10–7.15, (**b**) 7.15–7.20, and (**c**) 7.20–7.25. (**d**) AD and TD of CoCO_3_ prepared under different pH ranges.

The pH effect (7.10–7.15, 7.15–7.20, and 7.20–7.25) on the density of CoCO_3_ was investigated. In the low pH range (7.10–7.15), the initial particle sizes were quite high and the secondary particles found it quite difficult to regularly agglomerate (Figure 2a). Consequently, the sphericity of the final particle was not sufficient, resulting in a porosity increase during the accumulation of the secondary particles. Moreover, the apparent density and tap density were not relatively high. The experiment was also carried out under the pH range of 7.20 to 7.25 (Figure 2c), and the initial particles were quite tiny, presenting thin sheet shapes. The surface of the secondary particle was not sufficiently compact and the density of single particles was not sufficiently high, leading to very low tap density and apparent density values. However, the tap density and apparent density of the particles were the highest when the pH was retained in the range of 7.15 to 7.20 (Figure 2d). In Figure 2b, it can be observed that the accumulation mode of the primary particles was highly impacted and the sphericity of the secondary particles was quite good, with a round shape and high density, as well as low porosity following accumulation. 

#### 3.1.3. Effect of Feeding Speed on the Density of CoCO_3_

During the reaction, different feeding speeds could directly affect the growth speed, the amount of crystal seed, and the morphology of the crystal particles. The crystal particles could be dense and round, and impact each other under an appropriate growth speed; the highest AD and TD were obtained under such a scenario. In general, an excessively high growth of the radius would lead to small particle production, which could lower the density. Nevertheless, the radius of the primary particles would be too high to have sufficient density under the condition of a significantly slow feeding speed. 

**Figure 3:**
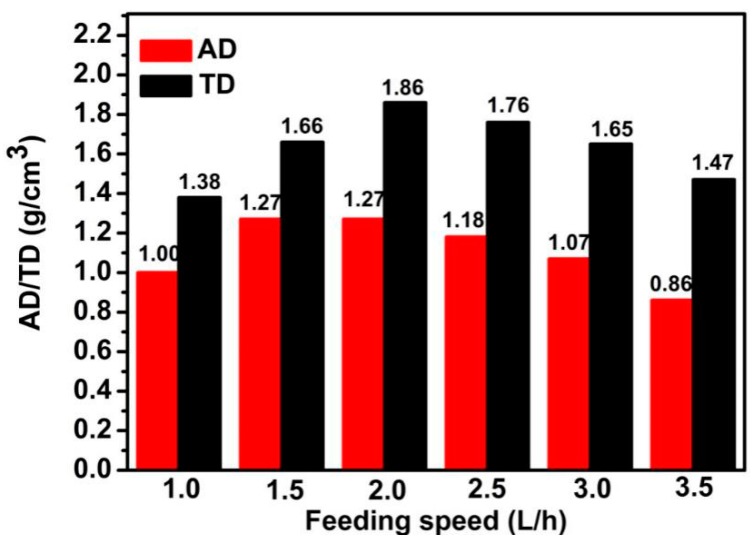
AD and TD of CoCO_3_ under different feeding speeds.

The AD and TD of CoCO_3_ under different feeding speeds are presented in Figure 3. When the feeding speed was lower than 2.0 L/h, the AD and TD increased along with the feeding speed increase. However, the AD and TD decreased along with the feeding speed increase when this value exceeded 2.0 L/h. Consequently, the AD and TD reached the maximum values when the feeding speed was 2.0 L/h, representing the best feeding speeds for the highest AD and TD during the crystallization.

**Figure 4:**
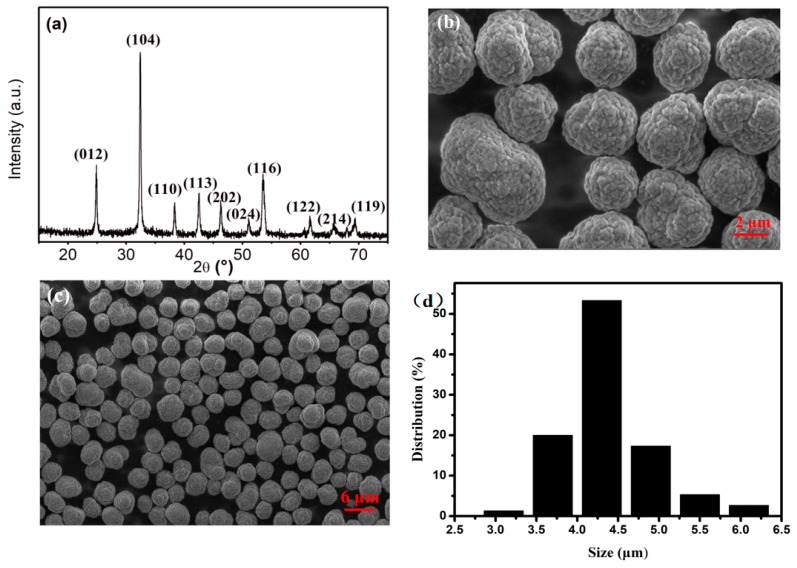
(**a**) XRD scan of the prepared CoCO_3_, (**b**,**c**) SEM images of the prepared CoCO_3_, and (**d**) particle size distribution of the prepared CoCO_3_.

In summary, the best reaction condition for CoCO_3_ preparation was: 60 g/L of NH_4_HCO_3_ as the base solution, pH retained within range of 7.15 to 7.20, and feeding speed of 2.0 L/h. The AD and TD of CoCO_3_ could reach the values of 1.27 g/cm^3^ and 1.86 g/cm^3^, respectively, when the reaction time was 23 h and the particle size was approximately 4.4 µm. The specific characterization results are presented in Figure 4.

Figure 4a presents the XRD patterns of the prepared CoCO_3_. All observed reflections belonged to the standard CoCO_3_ crystal structure (space group $R\bar{3}c$, JCPDS NO. 78-0209), hexagonal structure, with the parameters of a = 4.633 Å, b = 4.633 Å, c = 14.998 Å, α = 90°, β = 90°, γ = 120°. None of these observed peak reflections belonged to any other crystal, signifying a pure phase of the prepared CoCO_3_. Also, the sharp peaks in the XRD patterns indicated a good degree of crystallinity. Figure 4b shows the SEM image of the prepared CoCO_3_. It can be observed that the sample was spherical and its surface was round and accumulated as layered particles. Also, the sample presented a very good dispersion, as presented from the low-magnification SEM image (Figure 4c). Furthermore, the particle size was uniformly distributed between 4.0 µm and 4.5 µm, which was consistent with the measured result of 4.4 µm made using the composite laser particle size analyzer.

### 3.2. Growth Mechanism of the Continuous Reaction of CoCO_3_

#### 3.2.1. Crystal Anisotropy Growth

**Figure 5:**
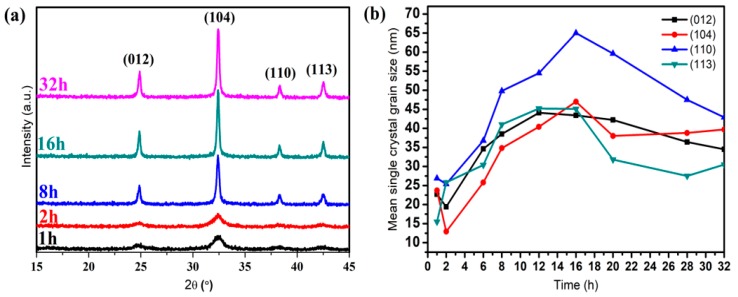
(**a**) XRD scan of the prepared CoCO_3_ under different crystallization times. (**b**) Average crystal size of different facet orientations ((012), (104), (110), (113)).

The crystal growth morphology depended on the relative growth rate of each crystal plane [28]. In Figure 5a, the XRD data of CoCO_3_ under different crystallization times are presented, where all these particles were assumed to be pure phases, indicating hexagonal structures. For the CoCO_3_ after 1 h and 2 h of crystallization, the peak intensity was very low and the peak shape was very broad, meaning the crystallinity of CoCO_3_ at the beginning was quite low, indicating an initial nucleation step. When the nucleation time increased to 8 h, the reflection peak became sharper, indicating that the crystallization of CoCO_3_ tended to be complete. The grain size calculated using the Scherrer equation (Equation (1)) based on each facet orientation is presented in Figure 5b. These grain sizes directly reflected the growth degree of the corresponding facet orientation groups [28]. In Figure 5b, the growth laws of grain size within the first 2 h of crystallization could not be observed. Subsequently, from 2 h to 16 h, the grain sizes of the main facets, namely (012), (104), (110), and (113), presented gradual growth states. Among these crystal planes, the crystal size of the (110) facet orientation grew the fastest and reached the maximum size at 16 h, indicating that the (110) facet was the main growth orientation during the crystallization. However, the grain size of all the facets presented a decreasing tendency up until 16 h of crystallization. This could be explained by the fact that all the facets broke at 16 h due to the collisions among crystal particles and the wall of the reactor under high speed stirring when the normal distance of each facet reached the critical value [29,30,31]. 

#### 3.2.2. Three-Dimensional Growth Model of Primary Particles 

**Figure 6:**
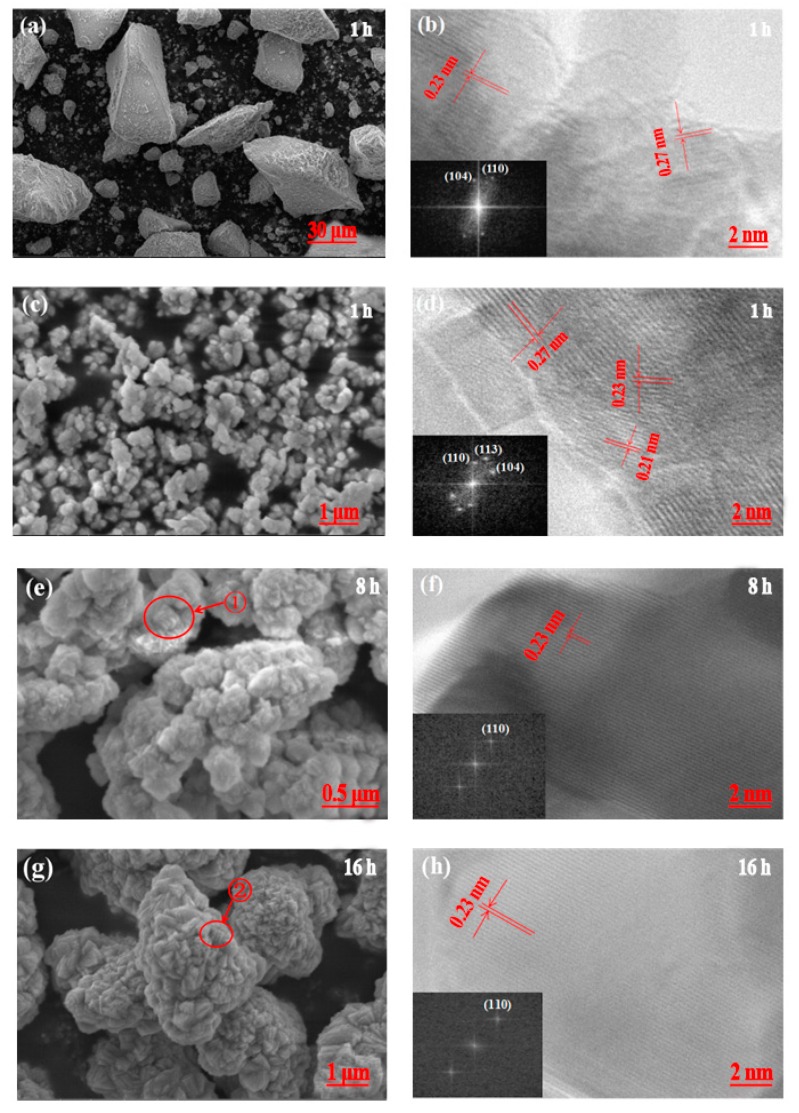

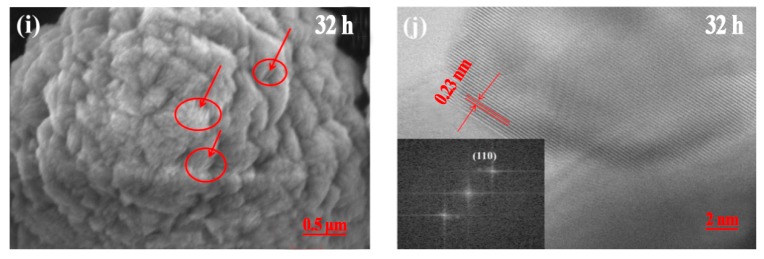
SEM images (**a**,**c**,**e**,**g**,**i**) and HR-TEM images (**b**,**d**,**f**,**h**,**j**) of prepared CoCO_3_ under different crystallization times: 1 h (**a**,**b**); 2 h (**c**,**d**); 8 h (**e**,**f**); 16 h (**g**,**h**); 32 h (**i**,**j**). Inset of (**b**,**d**,**f**,**h**,**j**): fast Fourier transformation (FFT) pattern of particles.

The SEM and HR-TEM images of the as-prepared CoCO_3_ under different crystallization times are presented in Figure 6. At the beginning of crystallization, the CoCO_3_ underwent soft agglomeration, forming irregularly shaped particles (Figure 6a). The determined d-spacing derived from HR-TEM of 0.27 nm and 0.23 nm was consistent with the layer spacing in the (104) and (110) orientations (Figure 6b). Then, the small particle agglomerates formed at the primary stage of nucleation, which can be observed in Figure 6c. These small particle agglomerates exposed the (104) facet with a d-spacing of 0.27 nm, the (110) facet with a d-spacing of 0.23 nm, and the (113) facet with d-spacing of 0.21 nm, indicating that none of these three facets had an absolute growth advantage (Figure 6d). 

Figure 6e presents the small sheet morphology of the primary particles and the non-compact sphere morphology of the secondary agglomerate particles. The corresponding HR-TEM image (Figure 6f) presents the (110) facet with a d-spacing of 0.23 nm. However, the (104) and (113) facets disappeared after being exposed at a nucleation time of 2 h, which signified that the primary particles were arranged in a relatively regular manner and the growth speed of the particles had a high preference for the normal direction of the (110) facet orientation. The particles kept agglomerating during the crystallization from 8 h to 16 h. The red circle marked in the SEM image shows that the primary particle seems to have increased (Figure 6g). The HR-TEM image, presented in Figure 6h, also shows only the (110) facet orientation. After crystallization for 32 h, the secondary particles agglomerated into compact sphere shapes and the exposed primary particles had a similar particle size compared to those at 16 h, which was consistent with the conclusion drawn from Figure 5b. Moreover, the HR-TEM image only presents the (110) facet, indicating that the (110) facet had an absolute growth advantage and this advantage could inhibit the growth of the other facets after 8 h. It appears that the grain size of the primary sheet-shaped particle gradually increased. It is worth being mentioning that the FFT of these particles strongly supports the d-spacing results from the HR-TEM images.

**Figure 7:**
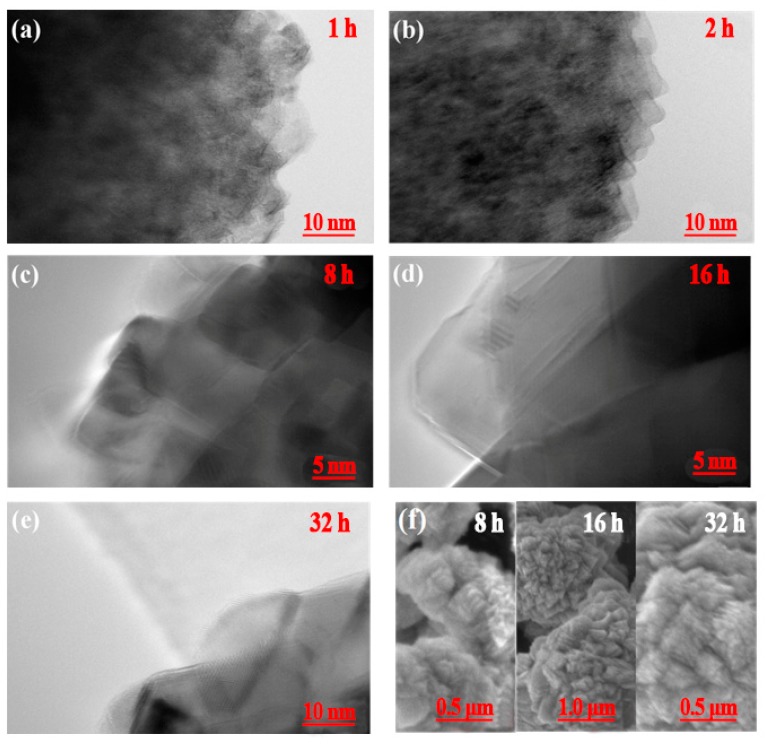
HR-TEM images (**a**–**e**) of the outer layers of CoCO_3_ particles for different crystallization times. (**f**) Partial enlargement SEM image of the area marked in Figure 6 (**e**,**j**,**i**) with a red cycle.

**Figure 8:**
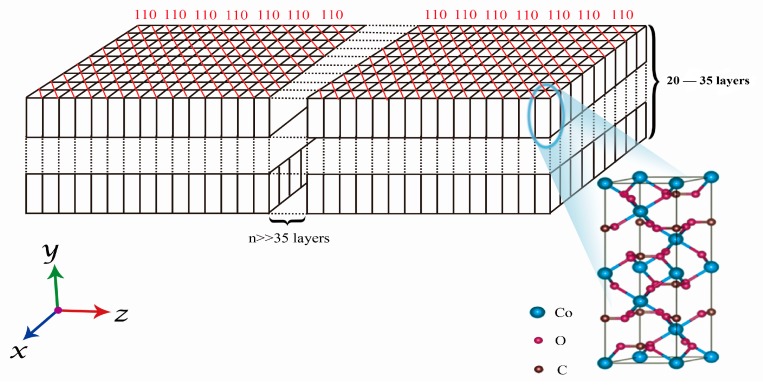
Growth diagram of the primary particle in three dimensions.

Figure 7 presents the HR-TEM images of the as-prepared CoCO_3_ for the crystallization times from 1 h to 32 h. It can be clearly observed that the outer primary particles were the sheet-shaped particles. Following 8 h of crystallization (Figure 7f), the outer particles still retained the sheet shape, which indicated that the primary particle gradually grew along the normal direction of the (110) facet orientation according to the comparative analysis of lattice plane and the HR-TEM image of Figure 6. In order to clarify the growth model of the primary particles in the three dimensions, the thickness of the sheet-shaped particles was measured to be 30–50 nm, as presented in Figure 6g,i with a red circle, along the normal direction of the (110) plane. The measured thickness was along the direction of the (110) plane, being the direction of the c-axis. The width along the c-axis of the unit cell of the CoCO_3_ was 1.5 nm, which signified that the sheet-shaped CoCO_3_ primary particles were arranged as 20–35 layers of the CoCO_3_ unit cell along the c-axis direction. In summary, the primary particles grew along the normal direction of the (110) plane in the horizontal direction and along the (001) plane in the width direction, forming the sheet-shaped particles. The growth diagram of the primary particle in the three dimensions and the corresponding unit cell of CoCO_3_ are presented in Figure 8.

#### 3.2.3. Agglomeration of Secondary Particles

The secondary particles were formed by the agglomeration of the primary particles. During the reaction, the collision rates among the particles would increase with the increase of the primary particles. Furthermore, the primary particles would be adsorbed by each other in order to lower the surface energy due to the smaller particle size, leading to a high surface energy. The initial adsorption was the simple physical bond adsorption, while the solubility was quite different at different positions of the large particles via adsorption. At the intersection part of the agglomerate, the curvature radius was negative. Consequently, the solubility of this part was calculated using the Kelvin equation (Equation (4)), indicating a lower solubility compared to the smooth crystal surface [32]:

(4) $$ln\frac{S}{S_{0}}=\frac{2\sigma }{\rho_{L}}\frac{M}{\rho RT}$$

where, *S* and *S*_0_ are the solubility values for a constant radius of curvature and a flat surface, respectively; *σ* is the average interfacial tension of each crystal face; *M* is the molecular molar mass of the crystalline material; *R* is the thermodynamic constant; *T* is the absolute temperature of the reaction system; and *ρ* is the real density of the crystalline material.

**Figure 9:**
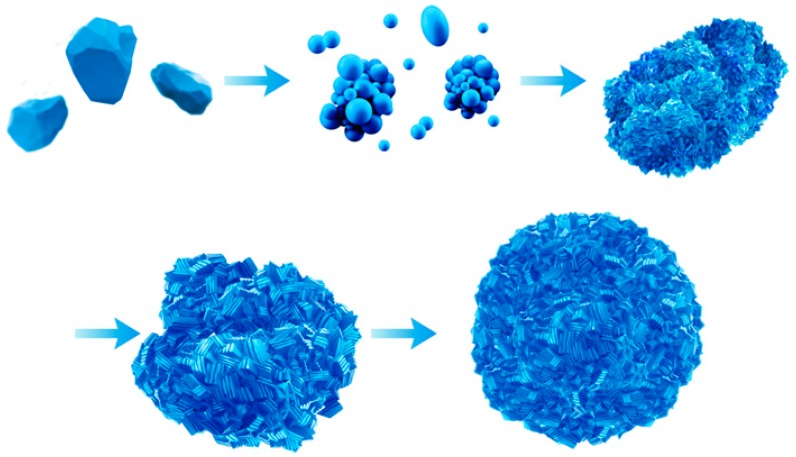
Agglomeration diagram of secondary particles.

According to the Thompson–Gibbs equation (Equation (3)), the supersaturation of crystal crystallization would increase with the solubility decrease under the reaction condition of a certain temperature and solution concentration, and the increase of supersaturation led to the decrease of the critical radius. Subsequently, the crystal would re-crystallize at the intersection where the primary particles bonded. Through this re-crystallization, the secondary particles grew significantly, while the gap among the primary particles gradually decreased. Finally, the particles grew to be compacted and round. The SEM images of the crystal following different crystallization times, as presented in Figure 6, supports the agglomeration model presented in Figure 9.

## 4. Conclusions

Through continuous carbonate precipitation, high density (1.27 g/cm^3^ and 1.86 g/cm^3^ for apparent density and tap density, respectively) and small particles size (4.4 µm) of CoCO_3_ were achieved under the reaction condition: 60 g/L for the concentration of NH_4_HCO_3_, 7.15–7.20 for the pH, and 2 L/h for the feeding speed of the CoCl_2_. The SEM and high-resolution TEM images demonstrated that the (110) facet grew the fastest during the crystallization and the primary particle of the CoCO_3_ had a sheet-shaped morphology. Furthermore, the horizontal direction of the sheet particle was in the normal orientation to the (110) facet of the CoCO_3_ unit cell, while the thickness direction of the sheet particle was in the (001) facet orientation of the CoCO_3_ unit cell. In addition, the thicknesses of the primary particles accumulated along the c-axis by 20–35 layers of the CoCO_3_ unit cell. The sheet-shaped particles transformed into the secondary spherical and compact particles through the agglomeration during the re-crystallization.
